# Ultraviolet Radiation on the Skin: A Painful Experience?

**DOI:** 10.1111/cns.12444

**Published:** 2015-08-30

**Authors:** Douglas M. Lopes, Stephen B. McMahon

**Affiliations:** ^1^ Neurorestoration group Wolfson Centre for Age‐Related Diseases King's College London London UK

**Keywords:** Inflammation, Pain, Pharmacology, UVB, UVR

## Abstract

Excessive exposure of skin to ultraviolet radiation (UVR) has dramatic clinical effects in humans, and it is a significant public health concern. Discomfort and sensory changes caused by skin sunburn are the main common features experienced by many of us, a phenomena triggered by the combination of long and short wavelengths radiation (UVA and UVB, respectively). Although the biological processes underlying UVR exposure are not fully understood, in the last few years many studies have made significant progress in characterizing sunburn at the cellular and molecular levels, making use of both humans and laboratory animal models. Here we review and reason that UVR can be used as an excellent model of sensitization and inflammation for pain research. UVR, particularly UVB, produces a controllable and sterile inflammation that causes a robust dose‐dependent hypersensitivity with minimal confounding effects. Importantly, we show that UVR animal models precisely recapitulate the sensory, cellular, and molecular changes observed in human skin, giving it great confidence as a translational model. Furthermore, in this article, we give an overview of the pharmacology underlying UVB inflammation, the latest advances in the field, and potential new targets for inflammatory pain.

## Introduction

Excessive exposure of the skin to ultraviolet radiation (UVR) is a common occurrence in tropical and even temperate latitudes. The result, sunburn, is well known to most of us. In extreme cases, this reaction can be life‐threatening 1, 2, but in most cases, it results in only a few days of discomfort. However, the sensory features of sunburn, which we review in this chapter, make this a very interesting experimental model to study, in a controllable way, features of the pain‐signaling system that are relevant to many forms of chronic pain. Specifically, we will argue that UVR can be used to selectively study the process of peripheral sensitization of nociceptors, with minimal confounding effects of any central sensory changes.

Recent epidemiological studies have demonstrated that sunburn is experienced by a large proportion of the population, affecting almost 75% of adolescents and young adults in the USA, and over 50% of the same age group in northern European countries 3, 4, 5, 6, 7. Despite its high prevalence, the understanding of the cellular processes underling the damages caused by UVR exposure remains limited, as are the treatments available to overcome the sensory changes associated with it. Developing and validating models to understand the biological processes implicated in sunburn offers the opportunity for improving our understanding of pain mechanisms. Most importantly, we reason in this review that UVR can be used as a model of inflammatory pain for a broad spectrum of studies in pain research in both humans and in laboratory animals.

## Consequences of UVR on Skin

Acute exposure to UVR triggers several changes in the skin. These include hyperemia, hyperalgesia, and inflammation, and all can result from exposure to different UV wavelengths. Notably, considerable evidence suggests that long‐wave UVA, which penetrates to the deeper layers of the dermis, has a relatively milder impact on the skin than short‐wave UVB irradiation, which is mainly absorbed by the epidermis 8. Histopathological studies dating back to the late 1970s tried to understand in more detail the effect of UVR on the skin. They revealed that UVA is more detrimental to deeper layers of the skin than UVB, affecting mainly capillaries by inducing degeneration of endothelial cells 9, 10, 11; however, this difference is to some extent dose‐dependent. Increasing the energy of the UVA irradiation also induces skin erythema 11, 12, but it is likely that cellular responses are distinct for differing wavelengths of UVR 9, 10, 11. Following these findings, a study evaluating hyperalgesia and erythema following UVA exposure suggested there are no major alterations in thermal or mechanical algesia either 1 or 24 h postirradiation 8. Furthermore, although UVA irradiation is sufficient to produce tanning of the skin, changes in erythema and skin temperature in the areas exposed to UVA were only observed for a few hours after exposure 8, 13. Although the authors concluded that UVA produced very limited hyperalgesia, very low doses of UVA (16.8 and 36 mJ/cm^2^) were used in these studies. A more recent study, however, comparing solar simulated radiation to UVA at similar erythema doses, the minimal UV dose sufficient to cause acute redness of the skin, demonstrated that both types of radiation produced similar skin sensitization 14. Additionally, a time course analyses demonstrated that 24 h after exposure a substantial increase in sensitivity to mechanical and heat pain was observed in the UVA irradiated area 14. Notably, the authors emphasized that the UVA doses used in the study (on average 56.5 mJ/cm^2^) are higher than those obtained from sunlight, which accounts for a minimal part of the solar radiation (approximately 10%) 14. Nonetheless, the study demonstrates that UVA can produce erythema, which is accompanied by tenderness of the skin and hyperalgesia, which peaks at 24 h postirradiation 14. At the opposite end of the spectrum sits UVC radiation, which, interestingly, has virtually no contribution to sunburn‐associated skin damage, as most of it is absorbed by the ozone layer and does not reach the Earth's surface 15, 16, 17. Together, these studies indicated that UVA does indeed induce skin changes; while UVA, UVB, and UVC can all induce erythema and sensory changes, the relative efficiencies of each of the wavelengths in causing damage and their abundance in solar radiation reaching the Earth's surface mean that UVB is responsible for most of the sunburn that humans naturally experience.

**Figure 1:**
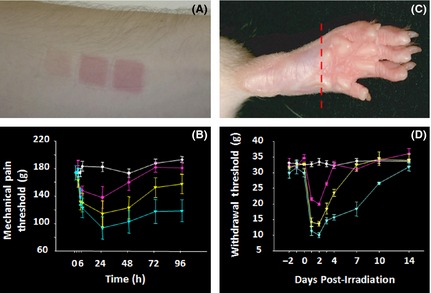
Features of UVB‐induced inflammation. (**A**) Cutaneous UVB exposure produces dose‐dependent erythema. From left to right patches of skin were exposed to 1, 2, and 3 MED on the volar aspect of the forearm 24 h previously. (**B**) Time course of UVB‐induced mechanical hyperalgesia in human volunteers. The white line shows sensitivity of a control site, while the red, yellow, and blue lines show changes after 1, 2, and 3 MED exposure, respectively. (**C**) Erythema in rat paw, 24 h after exposure of 500 mJ/cm^2^. (**D**) UVB‐induced mechanical hypersensitivity in rats, before and after exposure to 0 (white line), 250 mJ/cm^2^ (red), 500 mJ/cm^2^ (yellow), and 1000 mJ/cm^2^ (blue). These doses are roughly equivalent to 1, 2, and 4 MED.

It is well established that UV light in the UVB range is absorbed by the epidermis, leading to changes in its structure, and ultimately triggering cellular toxicity in the damaged region 11, 12. UVB irradiation leads to a range of intracellular changes, including DNA damage, changes in gene expression, increased levels of reactive oxygen species, and a significant inflammatory response at the injury site 18, 19, 20, 21, 22. More importantly, this phenomenon is accompanied by erythema and increased cutaneous hypersensitivity to mechanical and thermal stimuli (Figure 1A and B) 8, 13, 23, 24, 25. Furthermore, inflammation and hypersensitivity is reported to show both dose and time‐dependence peaking 24 to 48 h after the UV insult (Figure 1B) 8, 23. When comparing skin exposed to different spectra of UV, many studies have demonstrated that skin is most sensitive to UVB wavelengths and that these are most likely responsible for the erythema and altered sensory changes observed at the exposed area 8, 14, 23. An important characteristic of UVB is that it produces very similar changes in the skin of many mammals; in particular, sensory changes are similar in human, rat, and mouse skin (Figure 1C). This, as we will discuss, has led to its use as a translational model for the study of pain.

Wavelength is one variable that changes the ability of UVR to induce erythema and hyperalgesia in skin. Another variable is the pigmentation of skin, which, of course, can vary dramatically between individuals. For this reason, when UVR is administered to humans, it is usually “calibrated” in terms of its efficacy. The standard measure is MED or minimal erythemic dose; this is the amount of UVB, of any wavelength or mixture of wavelengths, that produces in an individual a clear area of erythema with distinct edges, as assessed 24 h after irradiation. Because laboratory animals are more homogeneous in their responsiveness to UVR, experimental studies that utilize animals often define the UV dose in terms of energy of irradiation.

UVB elicits sensitization of nociceptors. The skin is richly innervated by highly specialized sensory fibers that provide information to the CNS about the environment and integrity of the tissue 26, 27. Sensory receptors innervating the skin have been extensively studied. These fibers can be classified in many ways, but most traditionally by size, where three types of fibers are recognized: large myelinated A*β*, small myelinated A*δ*, and unmyelinated C fibers, each with a differential but overlapping sensitivity to applied stimuli 27, 28, 29, 30. However, we can generalize (at the risk of oversimplifying) to say that A*β* fibers mostly respond to innocuous mechanical stimulation, whereas A*δ* encode some forms of nociceptive stimuli as well as cold stimuli, C fibers can respond to noxious, warm, or innocuous mechanical stimuli 27, 28, 29, 30. The specific role of different classes of afferent fiber remains an area of active ongoing research. For our purposes here, the majority of A*δ* and C fibers are nociceptive and we are interested in how UVR affects their responsiveness.

Chronic UVR exposure can have effects beyond nociceptor sensitization. It is clear that sunlight is an essential part of human life, for example, it is required for the production of vitamin D; however, mounting evidence suggests there are long‐term damaging consequences to the skin. Regular exposure to solar radiation can lead to the development of deep wrinkles, leathery skin, dark spots, and dilatation of superficial blood vessels, collectively a process known as photoaging 31, 32, 33. Further to the cosmetic changes to the skin, an overwhelming number of scientific studies and epidemiological analyses have demonstrated that chronic exposure to the UV component of sunlight can lead to melanoma and nonmelanoma skin cancers 34, 35. Interestingly, more recent studies suggest that intermittent burning doses of UV during childhood are a major risk factor to develop skin cancer later in life 34, 36, 37. In addition, and contributing to its carcinogenic potential, chronic exposure to UV radiation can alter immune responses 38, 39, 40; this occurs via direct modulation of the immune system, creating a net imbalance toward immunosuppression 38, 39, 40. Although it is believed that UV‐induced immunosuppression is a transient protective process used by cells to repair and maintain genomic integrity, studies have indicated that there is a clear association between the immune suppressive effects of UV and its carcinogenic effect 40. It should be noted, however, that photoaging and cancer are consequences of long‐term and repetitive exposure to UV solar radiation, rather than a distinct feature of acute pain and hyperalgesia that is the focus of this review.

## UVB and Pain in Rodents: A Reliable Model

The importance of validating models that accurately reflect human diseases and/or conditions is a challenge in science. Although UVB‐induced hyperalgesia has been investigated in different species, from flies to humans 8, 14, 41, rodents are of course a particularly important preclinical model system. Aiming to study the consequences of UVB in rodents, Saade et al. irradiated the skin located over the back region of mice and observed a dramatic inflammatory response and changes in thermal sensitivity as consequence of UVB insult 42. Interestingly, these authors also showed a direct correlation between the UVB dose and decrease in the thermal sensitivity 42. Several subsequent studies confirmed the basic findings. Furthermore, following the work by Saade et al., there is a general consensus as to the time course of hyperalgesia: in most studies, it peaks between 24 and 48 h, after which the sensory changes slowly abate.

Just as in humans, local mechanical sensitization is observed in rodents exposed to UVB. One key study looked in greater detail at the skin damage observed upon acute exposure to UVR and successfully developed a model that reflects UVB‐induced inflammation and hypersensitization observed in humans 24. The study evaluated the plantar hind paw skin of rats exposed to different doses of UVB and demonstrated that increased thermal sensitization was induced in a dose‐dependent manner 24. Indeed, the authors showed that a single acute exposure of a 250 mJ/cm^2^ dose is sufficient to produce erythema and increased blood flow in the irradiated area 24. The range of doses evaluated in the study was sufficient to induce an increase in hind paw blood flow of up to 500%, together with a dramatic decrease in thermal pain threshold 24. Of equal importance, the study demonstrated a significant decrease (up to 80%) in the mechanical pain threshold in the area exposed to UVB (Figure 1D) 24. Interestingly, time course observations revealed sensory changes peaked 24 to 48 h postirradiation and coincided with the peak of erythema (Figure 1D) 24. Adding to these findings, Saade and collaborators further characterized this UVB model, by analyzing the skin at protein and cytokines levels, they revealed that inflammatory response occurs concomitantly with hypersensitivity 43. Consistent with previous observations, the decrease in pain thresholds triggered by UVB inflammation was reduced 72 h after exposure 24, 43. Additionally, sensitization triggered by UVR was restricted to the exposed paw only 43. Together, these key studies successfully developed a unique model that helped with the understanding of UVB‐mediated pain, as discussed below.

## What is the Mechanism of Hyperalgesia in UVB Irradiation: Peripheral and Central Sensitization?

It is well recognized that sensitization of the pain‐signaling system can arise at peripheral terminals (peripheral sensitization) or in the CNS, best studied in the spinal cord (central sensitization). Given the two distinct forms of alteration of pain processing, an obvious question is: What mechanism underlies UVR hyperalgesia? It is accepted that in some types of tissue trauma, increased sensitivity can also be seen in the tissues surrounding the injured area, a phenomenon believed to be a consequence of changes in neuronal excitability, particularly at the level of the spinal cord 44, 45, 46, 47.

Whether UVB injury induces changes in synaptic plasticity in the CNS is still a subject of debate in the pain field. Several lines of evidence from studies in humans demonstrate that increased mechanical and thermal sensitivity as a consequence of UVR damage is restricted to the site of irradiation and does not lead to central sensitization 14. Adding to this argument are data from a study directly comparing UVB inflammation to two other traditional models of hyperalgesia in human skin 23. Here, the authors demonstrated that whereas thermal burn and topical capsaicin produce both primary and secondary hypersensitivity (demonstrated by pin prick hyperalgesia and allodynia in adjacent areas of the lesion), in the UVB model skin sensitization is restricted to the inflamed area, without evoking changes in central pain processing 23; a similar conclusion has been reached by other research groups 48, 49, 50. One of these studies reports no changes in sensitization to heat, sharpness, or pressure at the secondary areas tested, indicating there had been no induction of central mechanisms underlying the UVB burn 50. Together, these studies suggest that peripheral sensitization is the predominant mechanism underlying UVB‐induced hyperalgesia in humans. However, there is some conflicting literature which we discuss later in the article.

Peripheral sensitization appears to be prominent in animal models of UVB irradiation. Although the first proposed animal model of UVB suggested that nonirradiated areas might be affected by UVR 42, perhaps due to the uncontrolled extent of the area damaged 42, Bishop and collaborators make a strong argument that UVB leads to a predominant peripheral sensitization 24. This study suggested that irradiation does not produce any spontaneous pain behavior in the area irradiated, such as flinching, licking, or paw lifting 24. More importantly, the group reports no induction of c‐fos immunoreactivity in the spinal cord of rats with irradiated hind paws compared to a sham irradiated group 24. The group did demonstrate that UVB irradiation can facilitate noxious‐evoked c‐fos expression at the spinal cord level, corresponding to the area irradiated, but attributed this to peripheral sensitization as there was no induction of c‐fos after UVB without noxious stimulation 24. Furthermore, it is well established that central sensitization is heavily dependent on recruitment of NMDA receptors 51, 52, 53, and it has been shown that UVB‐mediated mechanical hypersensitivity is not reduced by pharmacological blockade of spinal NMDA receptors 24. These experiments, together with other studies 43, provide strong evidence for an absence of spontaneous or ongoing pain induced by UVB, as is the experience of most people with sunburn. Furthermore, these findings set this model apart from other established models of inflammation, such as CFA and carrageenan, where central sensitization is clearly documented 53, 54, 55.

However, there are other somewhat conflicting studies. The first evidence suggesting that UVB induces sensitization in skin areas adjacent to inflammation comes from human studies 56. By evaluating the responses to different stimuli, the authors reported the occurrence of large areas of secondary pinprick hyperalgesia with increased sensitivity stable during a 10‐h follow‐up period after the first test 56. Notably, no differences in the heat and electrical pain tolerances were found at the secondary areas 56. In addition, a more recent study by the same authors reported only a small rim of dynamic mechanical hyperalgesia surrounding the sunburn area 57. Following these observations, studies from the same group reinforced the occurrence of central sensitization and proposed the use of different drugs to reduce the secondary mechanical hyperalgesia as result of UVB lesion 56, 58, 59, 60, 61. In these studies, relatively large areas of the skin were UVB‐burnt and were tested repeatedly with suprathreshold stimulation (which might itself induce central sensitization). These features may have induced spontaneous activity in nociceptors and can therefore explain the presence of central sensitization. Hence, differences in methodology may explain the contrasting results with the literature discussed previously.

Can UVB‐induced secondary hypersensitization be reproduced in animal models? Following the above observations in humans subjected to UVB burn, animal models of UVB‐induced hypersensitivity have also been investigated in the context of secondary sensitization 62, 63. Using the UVB rat model, Davies and colleagues proposed that, after injury, the area adjacent to the irradiation becomes sensitive to brush and punctate stimuli 62, although no changes in the threshold were observed on the side contralateral to the irradiation 62. The same study also investigated whether secondary hyperalgesia could be enhanced by the heat rekindling model, where a strong thermal stimulation is applied at the UVB‐irradiated area for a given period of time 62. Using this protocol, the authors reported that they can readily induce central sensitization in areas adjacent to the UVB burn, but this is then extended to the side contralateral to that irradiated 62. Furthermore, enhanced secondary skin hyperalgesia and allodynia were reported to be a long lasting event, continuing for up to 10 days after the insult 62. More recently, using an identical UVB+ heat rekindling model in combination with pharmacological manipulations, the same group replicated their findings, suggesting the occurrence of central sensitization and proposing the UVB+ heat rekindling as a translational model for inflammatory pain 63. It is difficult, however, to reconcile all these studies, as it appears that some paradigms promote primarily peripheral sensitization while others can also induce central changes.

## UVB‐Induced Hypersensitivity: Pharmacology

The pharmacological sensitivity of the UVR‐induced sensory changes is of interest in defining the utility of the model. Given the well‐acknowledged inflammatory changes and secretion of a great number of inflammatory mediators as a consequence to UVB exposure 18, 19, 42, it is not surprising that several studies have examined the effects of steroids on sunburn (Table 1). One of the first double‐blind controlled trials evaluating the effects of antiinflammatory drugs in patients demonstrated that oral administration of prednisone, either before or after UVB irradiation, does not decrease redness, edema or tenderness of the affected site 64. Since this first report, controlled trials have multiplied and similar drugs have been tested via systemic administration; however, they have had little apparent benefit to the subjects. For instance, a recent study demonstrated that 4 consecutive days of oral corticosteroid do not have any effect on erythema of irradiated skin 65. These studies provide clear evidence that systemic use of corticosteroids is not an effective treatment to alleviate the symptoms of skin sunburn.

**Table 1:** Summary of pharmacological interventions that have been tested in UVB models

Drug	Dose/Time	Via	Model	Outcome	Refs.
Corticosteroid					
Prednisone	80 mg (pre, during, and/or after irradiation)	Oral	Humans	No apparent benefit	64
Prednisone	30 mg for 4 days	Oral	Humans	No change in threshold erythema response	65
Dichlorisone, prednilosone ± dexamethasone or prednisone	Up to 5 mg, regularly to up to 4 days	Topical ± Oral	Humans	Variable effect, topical administration just as efficient as when used in combination with oral dosages	66
Hydrocortisone and other potent corticosteroid creams	Before and/or 1 and 4 h postirradiation	Topical	Humans	Hydrocortisone had no effect (unless applied before irradiation), whereas potent corticosteroids decreased erythema	67
Clobetasol propionate and hydrocortisone	Immediately after irradiation	Topical	Humans	Decreased erythema and pigmentation	70
Betamethasone dipropionate	Immediately after irradiation	Topical	Humans	Considerable reduction in erythema and blood flow from 24 h to 96 h	68
Methylprednisolone acetonate milk or hydrocortisone	0.1% solution, twice daily, during 7 days, starting 6 h after irradiation	Topical	Humans	Both drugs efficaciously reduced erythema, itch, and pain scores	69
Nonsteroidal anti inflammatory drugs (NSAID)					
Ibuprofen or k‐opioid receptor agonist	600 mg and 7.5 mg, respectively, single dose	Oral	Humans	Ibuprofen significantly reduced mechanical and heat hyperalgesia; k‐opioid had no apparent benefit	13
Ibuprofen	Single 800 mg dose, 22 h after irradiation	Oral	Humans	Ibuprofen significantly reduced mechanical and heat hyperalgesia as well as pain tolerance	71
Ibuprofen	Single 600 mg dose	Oral	Humans	Reduced erythema and heat pain threshold, with no much change in skin temperature	72
Remifentanil and/or gabapentin	Singles 0.08 ug/kg Remifentanil and/or 600 mg gabapentin doses	Oral	Humans	Remarkable effect of remifentanil (increased almost 90% heat pain tolerance threshold), whereas gabapentin did not show any positive effect	56
Rofecoxib (Cox‐2 selective inhibitor)	50, 250, or 500 mg, 24 h after irradiation	Oral	Humans	Reduction in heat pain perception and tolerance, as well as in secondary hyperalgesia	61
Ketorolac	2 mg	Intrathecal	Humans	Reduced areas of allodynia, when UVB was combined to HR and data analysed in a special manner	49
Indomethacin	2.5% solution, immediately after the exposure	Topical		Reduced skin temperature and hyperalgesia in the area exposed; no benefits of extra application	73
Indomethacin	1% cream, immediately after irradiation	Topical	Humans	Reduced erythema	70
Ibuprofen	Single, 50, 100, 200 mg/kg; or 0.215 g	Injected or topical gel	Humans and guinea‐pigs	Considerable reduction in thermal hyperalgesia and mechanical allodynia	24
Diclofenac	0.1% to 1%, gel	Topical	Humans	Effective on pain and burning sensation, reduced erythema, oedema, and skin temperature. Second application prolonged the beneficial effects of the drug.	74
Opioids					
Morphine or loperamide	Single dose, 1, 2, and 4 mg/kg	Injected	Humans	Reduction in thermal hyperalgesia and mechanical allodynia	25
Morphine or buprenorphine	Single doses, from 0.1 to 0.4%	Topical application	Humans	No effect on inflamed skin	79
Buprenorphine or fentanyl	Transdermal patches at 20 ug/h (for 144 h) and 25 ug/h (for 72 h), respectively	Local dermal patches	Humans	Buprenorphine, but not fentanyl, showed analgesic effects against pain. Adverse effects were reported.	48
Morphine or oxycodone	Single dose, 20 to 40 mg and 10 to 20 mg, respectively—immediately after irradiation	Oral	Humans	Both drugs showed a rapid and sustained antinociceptive and analgesic effect, particularly at the higher doses	78
Alterative targets					
NGF sequestering (TrkAd5 molecule)	Single dose, 2 mg/kg, subcutaneously, at the time of the inflammation	Injected	Rodents	Attenuation of thermal and mechanical hypersensitivity	24
TRPV1 antagonist SB‐705498	Single dose, 400 mg postirradiation	Oral	Humans	Increased heat pain tolerance and reduced flare area at the inflamed site. Some collateral effects were reported	87
TRPV1 antagonist ABT‐102	Single dose, 0.5, 2, and 6 mg, postirradiation	Oral	Humans	Reduced evoked pain at 2 and 6 mg doses	88
TRPV4 antagonist GSK205	Single application, 1 to 5 mM, pre‐irradiation	Local	Rodents	At the highest dose, there was an increase in the thermal threshold and striking elimination of tissue damage	89

Despite the negative results provided by studies using oral corticosteroids, other work suggests that topical application of steroids can be more effective (Table 1). Evidence from almost 50 years ago suggests a decrease in the discomfort of severe sunburn when aerosol corticosteroids were regularly applied in the affected area after UV overexposure 66. Following these observations, another study evaluated the effects of a variety of topical drugs applied to the skin of subjects exposed to UVB 67. The authors report that only potent corticosteroids were efficient in reducing erythema when applied after irradiation 67, with less convincing results when the skin was pretreated with antiinflammatory creams 67. Nevertheless, more positive outcomes with steroid creams after UVB sunburn were shown by further studies, which demonstrated a significant reduction of erythema and blood flow in irradiated skin treated with topical steroidal cream 68 or solutions 69. Furthermore, using a more refined method to evaluate skin damage after UVB exposure followed by topical application of steroid creams, an elegant study analyzed darker skin that was subject to different doses of UVB 70. The results confirmed the efficiency of corticosteroid in the alleviation of the erythema experienced, and interestingly, the authors demonstrated the suppression of pigmentation in the treated area 70. These authors reported a clear correlation between the degree of erythema and pigmentation, both of which could be suppressed if corticosteroid creams were applied immediately after UVB exposure 70. Regrettably, none of the cited studies evaluated any specific changes in the mechanical and heat threshold on the trial groups after the treatment presented above. However, it can be reasoned that use of topical corticosteroids is beneficial to alleviate UVB‐triggered pain while systemic corticosteroids are less so, presumably because of limitations in dosing.

UVB‐induced hypersensitivity can be alleviated by nonsteroidal antiinflammatory drugs (NSAID). A number of studies demonstrate the efficacy of systemic NSAID in reducing skin hyperalgesia to different stimuli 13, 24, 61, 71 (Table 1). For instance, it has been reported that single dose of ibuprofen can dramatically reduce the mechanical and heat sensitivity in the irradiated area 13. This analgesic effect of ibuprofen has been further validated by other studies 71, 72, which also reported a significant increase in pain tolerance in subjects treated with the drug 71. Following this trend, other NSAIDs also appear to be equally effective for treating sunburn when administered systemically 24, 56, 61. In contrast to these findings, however, another study demonstrated that intrathecal delivery of a NSAID can reduce areas of allodynia when UVB irradiation is combined with heat stimulus, but has little effect on UVB‐mediated burns alone 49. This further suggests that peripheral effects of NSAIDs in UVR account for most, if not all, of their beneficial effects. Thus, studies support that local NSAID can be effective in alleviating UVB‐induced sunburn. Following previous observations as to successful effect of NSAID when applied locally to the affected area 70, 73, four decades later a study comparing the effects of ibuprofen when delivered systemically or topically, revealed that both treatment methods equally attenuate hypersensitive and allodynia 24. Adding to these observations, other studies not only confirmed the benefits of local application of NSAID on UVB burns 74, but also suggested no additional advantage in delivering the drug systemically.

Other analgesic drugs have beneficial effects in alleviating the effects of UVB‐induced sunburn. For instance, opioids, a major class and one of the most powerful analgesic drugs 75, 76, 77, can efficiently reduce the sensory abnormalities triggered by UVB, whether delivered systemically or locally 24, 48, 78 (Table 1). However, some conflicting results have been reported regarding opioids efficacy 79, perhaps because of suboptimal dosing.

In addition to these traditional analgesic agents, a number of other drug classes have been evaluated in UVR. One example is the nerve growth factor (NGF). Given the well‐established role of NGF in inflammatory pain 80, 81, 82 and that UVR‐exposed skin releases NGF 42, 83, 84, 85, it would be reasonable to think that targeting NGF could be an effective method to reduce UVB‐mediated pain. Indeed, based on this idea, Bishop and collaborators demonstrated that by sequestering NGF, a modest but significant reduction in the magnitude of UVB‐induced sensory changes could be observed 24. Yet, regarding the pro‐hyperalgesic mechanism related to NGF, much evidence suggests that acute NGF stimulation leads to enhanced responsiveness of TRPV1 receptors 82, 86. Therefore, selective targeting of TRPV1 receptors might also be an effective approach to blocking UVB‐induced inflammatory pain, and some work supports this idea. Chizh and colleagues reported that TRPV1 antagonists can modestly reduce both hypersensitivity and flare area resulting from UVB inflammation 87. Further to these observations, a more recent study confirmed the therapeutic effects of TRPV1 antagonists, demonstrating the antinociceptive and antihyperalgesic effects of a new selective drug to these vanilloid channels 88. Adding to the role of TRPV receptor family in UVB‐hypersensitivity, another study claimed that UVB induces expression of TRPV4 channels at the epidermis, eliciting a proalgesic effect 89. Interestingly, UVB‐mediated skin damage and hypersensitivity were attenuated in TRPV4 KO mice and those pretreated with TRPV4 antagonist 89. Given that TRPV4 is abundantly expressed in keratinocytes and other epithelial cells 90, 91, 92, 93, the authors emphasized the role of this subfamily of epidermal cation channel as a proalgesic mediator of pain 89. Together, these studies have demonstrated that by understanding mechanisms underlying UVB hyperalgesia and sensory changes during inflammation, new therapeutic approaches to treat inflammatory pain states in general can be developed. Further to the targets above discussed, new exciting strategies could be developed by the identification of cellular mechanisms triggered by pain, as discussed below.

## Changes in mRNA Transcription and Protein Levels after UVB Burn: New Potential Therapeutic Targets

Although the underlying mechanism of UVB‐mediated hypersensitivity is not entirely understood, much evidence suggests that the major component of sensitization lies on the afferent terminals at the skin 25, 94. Therefore, this model may allow us to identify novel mediators of sensitization that are important in the UVR and other pain states. Aiming to identify possible peripheral mediators of hyperalgesia in response to UVB burn, Dawes and collaborators took advantage of developments in “omics” to analyze an array of 90 different inflammatory mediator candidates in the skin of rats and humans that were subjected to UVB insult 94. A large number of upregulated transcripts were identified by the group, among them a variety of interleukins, chemokines, and cyclooxygenase and iNOS that were consistent between humans and rats 94. Notably, a dramatic upregulation of CXCL5 expression was observed at the peak of inflammation 94. Moreover, the authors demonstrated that injection of CXCL5 is proalgesic, producing a reduction of mechanical pain threshold, similar to UVB irradiation 94. Most importantly, the study showed that blockade of CXCL5 postirradiation, using a neutralizing antibody, reduces the mechanical pain threshold, as well as the levels of the immune response at the site 94. Recently, a further investigative study not only reproduced the data in the human skin, but also suggested that fibroblasts might be involved in triggering the expression and secretion of CXCL5 at the skin, ultimately boosting UV response 95. Given these findings, a step forward toward the validation of these targets is crucial, so they could meaningfully represent a plausible treatment for inflammatory hyperalgesia.

Other recent studies have identified unusual candidate molecules that might be involved in UVB‐inflammatory pain. One group used the same principle of UVB inflammation but with the intent of identifying lipid mediators 96. By comparing tissue from skin, DRG's and spinal dorsal horn, the study identified almost 20 different lipids isoforms that were upregulated upon UVB irradiation, nearly all in peripheral tissues and almost none at DRG and spinal cord levels 96. Importantly, among this new potential targets, five lipids (lipophosphatic acid 1:18 and 9; 13‐S‐hydroxyoctadecadienoic acid; 5 and 12‐hydroxyeicosatetraenoic acid), which were recently identified as TRPV1 agonists 96, 97, 98, were elevated in the skin of UVB rodents, suggesting that they might contribute to thermal hyperalgesia and mechanical allodynia observed in inflammation 96.

Another study examined molecular changes after UVB irradiation at the transcriptional level using RNA sequencing. By comparing skin samples of patients and rodents exposed to UVB, the authors presented a remarkable level of similarity across the different species, identifying changes on over 800 common genes 99. Most of the changes in expression were found in the skin, as previously reported 96. Unsurprisingly, many of the genes upregulated were related to inflammation 99. The group also looked at molecular changes at the DRGs and identified 39 genes differentially regulated 99. Interestingly, among the transcriptional changes, VGF, a NGF‐induced gene which has been implicated in driving abnormal pain behavior 100, 101, 102, was identified as one of the most upregulated genes 99. These correlation analysis and in‐depth molecular studies not only add an extra layer of reassurance to the validation of the translational model for UVB, but also provide new appealing targets with clinical relevance in the pain field.

## Conclusions

There is a considerable need to develop new classes of analgesic drugs 103, 104, 105, and as a result, there have been many efforts to bring new drugs to the clinic. Unfortunately, these efforts have, to date, had only limited success in terms of new drug registrations. There have been some successes, particularly in the last few years, with positive phase II trials in pain and related sensory disturbances using anti‐NGF, sodium channel blockers, and Angiotensin II receptor and P2X3 receptor antagonists 105, 106, 107, 108, 109, 110, 111, 112. One of the blocks to drug development in this area has been translation from preclinical studies to humans. There are probably multiple contributory factors, but one in particular has been a concern over some of the models of persistent pain used in the preclinical studies. We would like to propose here that UV‐induced sunburn may be a useful model to help in efforts to understand inflammatory pain and develop new drugs. The model produces a localized inflammatory response in an accessible tissue. It precipitates a reasonably well‐defined series of sensory changes, and particularly clear cut peripheral sensitization of nociceptors, a process likely to be of considerable clinical relevance in many pain states. Most importantly, the UVR model of inflammation can be elicited both in humans and laboratory animals, apparently with consistent features in these species. This last feature, which is not shared by many of the preclinical models used, allows for a much greater confidence in translation. As the model can be induced easily in humans, it can also be used in phase I studies and potentially provide an early indication of efficacy.

## Conflict of Interest

The authors declare no conflict of interest.
